# Investigation of association between *LINC00673* rs11655237 C>T and Wilms tumor susceptibility

**DOI:** 10.1002/jcla.22930

**Published:** 2019-07-01

**Authors:** Xiaofeng Gao, Wei Jia, Jinhong Zhu, Wen Fu, Shibo Zhu, Huimin Xia, Jing He, Guochang Liu

**Affiliations:** ^1^ Department of Pediatric Surgery, Guangzhou Institute of Pediatrics, Guangzhou Women and Children's Medical Center Guangzhou Medical University Guangzhou China; ^2^ Molecular Epidemiology Laboratory, Department of Clinical Laboratory Harbin Medical University Cancer Hospital Harbin China

**Keywords:** *LINC00673*, polymorphism, susceptibility, Wilms tumor

## Abstract

**Background:**

Wilms tumor (WT) is the most common pediatric renal malignancy. Previous genome‐wide association studies have identified that the *LINC00673* rs11655237 C>T polymorphism is associated with the risk of several types of cancer. However, few studies have investigated the association between *LINC00673* rs11655237 C>T and WT susceptibility.

**Method:**

We genotyped *LINC00673* rs11655237 C>T in 145 patients with WT and 531 cancer‐free controls recruited from southern Chinese children. The strength of association was estimated by odds ratios (ORs) and 95% confidence intervals (CIs).

**Results:**

Our study indicated that there was no significant association between *LINC00673* rs11655237 C>T polymorphism and WT risk under all the tested genetic models (CT vs CC: adjusted OR = 0.94, 95% CI = 0.63‐1.40; TT vs CC: adjusted OR = 0.60, 95% CI = 0.22‐1.59; TT/CT vs CC: adjusted OR = 0.89, 95% CI = 0.61‐1.31; and TT vs CC/CT: adjusted OR = 0.61, 95% CI = 0.23‐1.61). Further stratified analysis detected no significant association, either.

**Conclusion:**

In conclusion, we failed to find any association between the *LINC00673* rs11655237 C>T polymorphism and WT risk. This finding needs to be verified in larger studies and other populations.

## 1 INTRODUCTION

Wilms tumor (WT), an embryonal cancer, constitutes 95% of all renal cancers among children not more than 15 years old.^1^, ^2^ WT is the most common pediatric renal tumor, followed by clear cell sarcoma of the kidney. The incidence of WT in China is ~3.3 per million, lower than that in Western countries.^3^ Usually, the treatment regimen for WT contains nephrectomy and systemic chemotherapy. Nowadays, the overall survival rate of WT is around 90%, compared with 30% in the 1930s^3^, ^4^. The etiology of WT is not yet clearly known. It is believed that some alterations in certain genes associated with genitourinary tract development may predispose to WT. However, the molecular mechanisms for this differentiation failure in the tumor are still indefinite.

Childs et al^5^ examined the impacts of variation at 2p13.3, 3q29, 7p13, and 17q25.1 on cancer susceptibility. They showed that a rs11655237C>T polymorphism was significantly associated with pancreatic cancer risk; this polymorphism is located in a gene encoding a long intergenic non‐coding RNA (lincRNA) *LINC00673* at 17q25.1 region. The rs11655237C>T was the most significant polymorphism in that study. In addition, it was reported that rs11655237C>T in exon 4 of *LINC*00673 formed a target site for *miR‐1231* binding, consequently restraining the *LINC00673*'s tumor‐suppressing function and reducing the susceptibility of pancreatic cancer.^6^ Moreover, *LINC00673* is also associated with the risk of non–small‐cell lung cancer (NSCLC), gastric cancer, breast cancer, hepatocellular cancer, tongue squamous cell carcinoma, and neuroblastoma.^7^, ^8^, ^9^, ^10^, ^11^, ^12^, ^13^, ^14^ In total, these results suggest that *LINC00673* rs11655237C>T is implicated in a broad spectrum of tumors. Nevertheless, the association between *LINC00673* rs11655237 C>T polymorphism and WT susceptibility is not yet reported. So we enrolled 145 cases and 531 cancer‐free controls to evaluate the impact of *LINC00673* rs11655237 C>T polymorphism on WT risk.

## 2 MATERIALS AND METHODS

### 2.1 Study subjects

A total of 145 histopathologically confirmed cases of WT from Guangdong province (Southern China) were included in our study,^15^, ^16^, ^17^, ^18^, ^19^, ^20^, ^21^ along with 531 unrelated, age‐, gender‐, and race‐matched cancer‐free controls.^22^, ^23^, ^24^ Both imaging and pathological examination were used to diagnose the disease. All study objects were recruited from the Guangzhou Women and Children's Medical Center from March 2001 to June 2016. About 2 mL of peripheral blood was collected from each individual for genomic DNA extraction. We conducted this study with the approval of the Institutional Review Board of Guangzhou Women and Children's Medical Center. All participants offered informed consent signed by their guardians.

### 2.2 Genotyping

Total genomic DNA was extracted from peripheral blood leukocytes with the TIANamp Blood DNA Kit (TianGen Biotech Co.).^22^ Genotyping was conducted blind to the status of the case or the control. The TaqMan real‐time PCR was performed as previously described.^25^, ^26^, ^27^ Besides, approximately 10% of the DNA samples were randomly selected and re‐genotyped. The result of reproducibility was 100%.

### 2.3 Statistical analysis

The differences in the demographic and genotypic information between WT cases and controls were compared using chi‐squared test. The association between *LINC00673* rs11655237 C>T and WT susceptibility was evaluated by calculating the odds ratios (ORs) and 95% confidence intervals (CIs) with unconditional multivariate logistic regression analyses. *P*‐values < 0.05 were considered as statistically significant. All statistical analyses were two‐sided and performed using SAS software (Version 9.4; SAS Institute).

## 3 RESULTS

### 3.1 *LINC00673* rs11655237 C>T polymorphism and WT susceptibility

The demographic characteristics of participants were described in Table S1. The genotype frequencies of the *LINC00673* rs11655237 C>T polymorphism in WT cases and controls are shown in Table 1. There was no significant deviation from the Hardy‐Weinberg equilibrium in the controls (*P* = 0.279). Besides, we found that there was no significant association under all the tested genetic models (CT vs CC: adjusted OR = 0.94, 95% CI = 0.63‐1.40; TT vs CC: adjusted OR = 0.60, 95% CI = 0.22‐1.59; TT/CT vs CC: adjusted OR = 0.89, 95% CI = 0.61‐1.31; and TT vs CC/CT: adjusted OR = 0.61, 95% CI = 0.23‐1.61).

**Table 1:** Genotype distributions of *LINC00673* rs11655237 C>T polymorphism and Wilms tumor susceptibility

Genotype	Cases (N = 145)	Controls (N = 531)	*P* a	Crude OR (95% CI)	*P*	Adjusted OR (95% CI)^b^	*P* b
rs11655237 (HWE = 0.279)							
CC	92 (63.45)	325 (61.21)		1.00		1.00	
CT	48 (33.10)	178 (33.52)		0.95 (0.64‐1.41)	0.809	0.94 (0.63‐1.40)	0.763
TT	5 (3.45)	28 (5.27)		0.63 (0.24‐1.68)	0.357	0.60 (0.22‐1.59)	0.302
Additive			0.645	0.89 (0.64‐1.22)	0.459	0.87 (0.63‐1.20)	0.393
Dominant	53 (36.55)	206 (38.79)	0.622	0.91 (0.62‐1.33)	0.623	0.89 (0.61‐1.31)	0.561
Recessive	140 (96.55)	503 (94.73)	0.366	0.64 (0.24‐1.69)	0.371	0.61 (0.23‐1.61)	0.318

Abbreviations: CI, confidence interval; HWE, Hardy‐Weinberg equilibrium; OR, odds ratio.

a Chi‐square test for genotype distributions between Wilms tumor patients and controls.

b Adjusted for age and gender.

### 3.2 Stratified Analysis

Further stratified analysis by age, gender, and clinical stages was conducted (Table 2). The result showed no significant genetic association between the *LINC00673* rs11655237 C>T polymorphism and WT risk in the subgroups defined by age, gender, or clinical stages.

**Table 2:** Stratification analysis for the association between *LINC00673* rs11655237 C>T polymorphism and Wilms tumor risk

Variables	CC	CT/TT	Crude OR (95% CI)	*P*	Adjusted OR^a^(95% CI)	*P* a
	(Cases/Controls)					
Age, month						
≤18	41/143	25/90	0.97 (0.55‐1.70)	0.912	0.96 (0.55‐1.69)	0.896
>18	51/182	28/116	0.86 (0.51‐1.44)	0.571	0.85 (0.51‐1.43)	0.547
Gender						
Females	40/141	24/92	0.92 (0.52‐1.63)	0.773	0.91 (0.52‐1.62)	0.758
Males	52/184	29/114	0.90 (0.54‐1.50)	0.686	0.87 (0.52‐1.46)	0.597
Clinical stages						
I	1/325	3/206	4.73 (0.49‐45.81)	0.180	4.40 (0.45‐43.03)	0.203
II	28/325	21/206	1.18 (0.66‐2.14)	0.578	1.15 (0.63‐2.09)	0.641
III	35/325	15/206	0.68 (0.36‐1.27)	0.223	0.68 (0.36‐1.28)	0.228
IV	22/325	11/206	0.79 (0.38‐1.66)	0.532	0.79 (0.38‐1.68)	0.545
I + II	29/325	24/206	1.31 (0.74‐2.31)	0.358	1.27 (0.72‐2.26)	0.410
III + IV	57/325	26/206	0.72 (0.44‐1.18)	0.193	0.72 (0.44‐1.19)	0.201

Abbreviations: CI, confidence interval; OR, odds ratio.

a Adjusted for age and gender.

## 4 DISCUSSION

Our study firstly explored the relevance between *LINC00673* rs11655237 C>T polymorphism and WT susceptibility, and the result indicated that the *LINC00673* rs11655237 C>T polymorphism was not associated with WT in a southern Chinese population.

LincRNAs, as the largest subclass in the non‐coding transcriptome, are non‐coding transcripts longer than 200 nts. Like the miRNAs, LincRNAs also play important roles in human disorders, especially tumors, and exhibit distinct gene expression patterns in primary tumors and metastases^28^, ^29^. LincRNAs expression is strikingly tissue‐specific, when compared with coding genes.^30^ In the past decades, LincRNAs are known to play key roles in the process of imprinting, metastasis, deregulation of tumor suppressors, and pseudogene pairing.^31^, ^32^, ^33^, ^34^, ^35^, ^36^, ^37^ LincRNAs have potentials be widely used for cancer diagnosis, prognosis and served as potential therapeutic targets in the future. However, revealing the function of individual LincRNAs remains a great challenge for human beings.

*LINC00673* has been reported to be associated with susceptibility, progression, and outcome of some malignancies. A research by Childs et al^5^ showed that *LINC00673* is significantly associated with pancreatic cancer susceptibility. Moreover, *LINC00673* rs11655237 variant, a germ line C>T transition, can cause a down‐regulation of *LINC00673* in cells. Decreased level of *LINC00673* may lead to the activation of SRC‐ERK oncogenic signaling, but attenuation of the STAT1‐dependent anti‐oncogenic signaling. The study conducted by Shi et al^28^ showed that the oncogenic activity of *LINC00673* is partially attributable to the repression of NCALD through interaction with LSD1, an epigenetic repressor which promoted NSCLC progression. As a tumor suppressor gene, *HOXA5* inhibits NSCLC metastasis through regulating cytoskeletal remodeling. Ma et al^29^ firstly found that *LINC00673* promoted NSCLC metastasis by the binding of EZH2 and then epigenetically silencing *HOXA5*. A study by Lu et al^7^ demonstrated that *LINC00673* modulated cell proliferation, migration, invasion, and epithelial‐mesenchymal transition by sponging *miR‐150‐5p* and regulating *ZEB1* expression indirectly. Huang et al^9^ found that *LINC00673* was overexpressed in gastric cancer. Abdul‐Rahman et al^10^ found that *LINC00673* could be a prognostic marker in patients with breast cancer. Yu et al^13^ reported that the up‐regulation of *LINC00673* might lead to poor survival and promote metastasis in tongue squamous cell carcinoma.

Moreover, in our earlier research conducted by Zhang et al,^14^ we found that *LINC00673* rs11655237 C>T polymorphism was significantly associated with neuroblastoma susceptibility in Chinese Han population. Given the important role of *LINC00673* in malignancies, we conducted the current study regarding the association between *LINC00673* rs11655237 C>T and WT risk in southern Chinese children. We found that there was no significant association between *LINC00673* rs11655237 C>T and WT risk.

To the best of our knowledge, this is the first study to explore the correlation between the *LINC00673* rs11655237 C>T polymorphism and WT risk. Several limitations should be addressed. First, because of the low incidence rate of WT, the small sample size might limit the statistical power. Second, as a retrospective study, selection bias and information bias were inevitable. In the end, only one polymorphism of *LINC00673* was studied, and in the future, more potentially functional polymorphisms need to be explored for their association of WT risk.

In conclusion, our result indicated that there was no significant association between the *LINC00673* rs11655237 C>T polymorphism and WT risk in the southern Chinese population. In the future, well‐designed prospective studies that include larger sample sizes and different ethnics should be performed to validate our findings.
