# Supplementary material for: Investigation of association between LINC00673 rs11655237 C>T and Wilms tumor susceptibility
Source: J Clin Lab Anal. 2019 Jul 1;33(7):e22930. doi: 10.1002/jcla.22930 (PMC6757132; doi:10.1002/jcla.22930)
Supplement: Supplementary file 1 [file JCLA-33-e22930-s001.doc]

| **Supplemental Table 1**. Frequency distribution of selected variables in Wilms tumor patients and controls | | | | | |
| --- | --- | --- | --- | --- | --- |
| Variables | Cases (n=145) | | Controls (n=531) | | *P ^a^* |
|  | No. | % | No. | % |  |
| Age range, month | 1-132 | | 0.07-156 | | 0.725 |
| Mean ± SD | 26.17 ± 21.48 | | 29.73 ± 24.86 | |  |
| ≤18 | 66 | 45.52 | 66 | 45.52 |  |
| >18 | 79 | 54.48 | 79 | 54.48 |  |
| Gender |  |  |  |  | 0.956 |
| Female | 64 | 44.14 | 233 | 43.88 |  |
| Male | 81 | 55.86 | 298 | 56.12 |  |
| Clinical stages |  |  |  |  |  |
| I | 4 | 2.76 |  |  |  |
| II | 49 | 33.79 |  |  |  |
| III | 50 | 34.48 |  |  |  |
| IV | 33 | 22.76 |  |  |  |
| NA | 9 | 6.21 |  |  |  |
| SD, standard deviation; NA, not available.  ^a^ Two-sided *χ^2^* test for distributions between Wilms tumor patients and controls. | | | | | |
